# Combined Associations of Type 2 Diabetes and Peripheral Neuropathy With Device‐Measured Physical Activity and Sedentary Behaviour—The Maastricht Study

**DOI:** 10.1002/dmrr.70069

**Published:** 2025-07-22

**Authors:** Touria Ahaouari, Brenda Berendsen, Nicolaas Schaper, Hans Bosma, Marleen van Greevenbroek, Bastiaan de Galan, Miranda T. Schram, Hans Savelberg, Annemarie Koster

**Affiliations:** ^1^ Nutrition and Translational Research in Metabolism (NUTRIM) Maastricht University Maastricht the Netherlands; ^2^ Care and Public Health Research Institute (CAPHRI) Maastricht University Maastricht the Netherlands; ^3^ Cardiovascular Research Institute Maastricht (CARIM) Maastricht University Maastricht the Netherlands; ^4^ Department of Nutrition and Movement Sciences Maastricht University Maastricht the Netherlands; ^5^ Department of Internal Medicine Maastricht University Medical Centre Maastricht the Netherlands; ^6^ Department of Social Medicine Maastricht University Maastricht the Netherlands; ^7^ Department of internal Medicine Radboud University Medical Centre Nijmegen the Netherlands; ^8^ MHeNS School for Mental Health and Neuroscience Maastricht University Maastricht the Netherlands; ^9^ Heart and Vascular Centre Maastricht University Medical Center+ Maastricht the Netherlands

**Keywords:** accelerometry, peripheral neuropathy, physical activity, sedentary behaviour, type 2 diabetes mellitus

## Abstract

**Objective:**

Peripheral neuropathy (PN) is a common complication of type 2 diabetes mellitus (T2DM). In this study, we determined the independent and combined associations of T2DM and PN with device‐based measures of physical activity levels and sedentary behaviour.

**Materials and Methods:**

Cross‐sectional data from The Maastricht Study were used (*N*: 6471, age 59.8 ± 8.8). T2DM was determined with an oral glucose tolerance test and PN was, using a neurothesiometer, defined as an impaired vibration perception threshold (iVPT), that is exceeding 25 V in either one or both halluces. Physical activity and sedentary behaviour outcomes were derived through 8 days of activPAL accelerometer measurement, worn 24 h/day. Multiple linear regression analyses were used with adjustment for demographic, lifestyle and health‐related indicators.

**Results:**

In the fully adjusted model, the combined presence of T2DM and iVPT presented the lowest step count (−1407 steps/day [95% CI: −1851, −963]), and showed the lowest time in light‐intensity (−27.2 min/day [−38.6, −15.8]) and moderate‐to‐vigorous physical activity (−9.5 min/day [−12.6, −6.5]). Moreover, those with both conditions had the highest sedentary time (+33.3 min/day [21.4, 45.2]) and longest sedentary bout durations (+1.0 min/bout [0.6, 1.4) compared with those without these conditions.

**Discussion:**

T2DM and PN were both independently associated with lower levels of physical activity and higher levels of sedentary time. The combination of T2DM with PN was associated with particularly low levels of physical activity and higher levels of sedentary time, indicating an additive association. Strategies to improve physical activity in these individuals should address both conditions.

**Abbreviations**
iVPT: impaired vibration perception threshold
LIPA: light intensity physical activity
MVPA: moderate‐to‐vigorous physical activity
NGM: normal glucose metabolism
PA: physical activity
PN: peripheral neuropathy
preDM: prediabetes
T2DM: type 2 diabetes mellitus

## 1 Introduction

Peripheral Neuropathy (PN) is caused by the degeneration of small and large nerve fibres accompanied by impaired nerve regeneration that starts in the distal parts of the lower extremities [1]. PN is characterised by a combination of sensory neuropathy, motor neuropathy, and autonomic neuropathy [2]. Factors such as hyperglycaemia, advanced age, higher height, smoking, genetic factors, large waist circumference, and peripheral artery disease are associated with worsening peripheral nerve function [3, 4]. One of the common causes of PN is diabetes, but a variety of other conditions have also been associated with PN, such as toxins, inflammatory/immune diseases, vitamin B12 deficiency and inherited disorders [1]. Up to 50% of those diagnosed with type 2 diabetes mellitus (T2DM) exhibit signs and symptoms of PN, but reported prevalence data can vary markedly because of factors such as disease duration, level of glycaemic control and presence of other diabetic complications [5]. These factors contribute to the prevalence of PN in individuals with diverse characteristics, particularly in the presence of T2DM.

PN can cause a range of symptoms, such as changes in motor function and sensory perception. Whereas less is known about changes in motor function related to diabetic neuropathy, these alterations can affect small joints and muscles, resulting in muscle weakness, abnormal loading of the foot, and loss of balance [6]. The altered proprioceptive feedback in the lower extremities due to sensory neuropathy can result in decreased sensorimotor control [7], impaired body spatial positioning and reduced maintenance of balance and gait [8, 9]. Reduced sensory peripheral nerve function can be evaluated with a neurothesiometer, which measures the vibration perception threshold (VPT). Impairment of VPT indicates abnormal sensitivity to vibration, reflecting deficits in large fibre sensory nerves. Sensory symptoms—such as impaired sensation, tingling and pain—and the altered sensorimotor control are the main mediators of decreased quality of life and depression in individuals with sensory neuropathy [8, 10]. Sensorimotor alterations associated with PN are a major risk factor for falling, leading to fear of falling and limited engagement in daily activities [11, 12]. Moreover, sensory neuropathy can result in loss of protective sensation and the impaired perception in the feet has been linked to an increased risk of foot ulceration [13, 14].

Maintaining an active lifestyle is one of the central recommendations for the management of diabetes [15]. However, individuals that have both diabetes and PN may find it particularly challenging to maintain an active lifestyle due to the comorbidities and symptoms associated with both PN and diabetes. Nevertheless, physical activity (PA) should be encouraged as it has been shown to improve glycaemic control [16], reduce PN symptoms [17], and improve overall health status. There is relatively little research on the association between PN with PA and sedentary behaviour in individuals with and without T2DM. A nerve conduction study, performed in a selected group of older men (mean age 79 years) without diabetes, reported that worse peroneal motor latency (but no other electrophysiological measures) was associated with lower self‐reported PA and that worse sural amplitude was associated with slightly less daily vigorous activity [18]. Another study showed that individuals with both T2DM and PN had lower self‐reported PA than those without PN [19]. Among the few studies investigating PA and PN, only one relied on objective measures of PA, which are preferred over self‐reporting. Using a pedometer, individuals with T2DM and PN had a significantly lower step count compared with those without PN [20]. Additionally, sedentary behaviour, a distinct behavioural domain with independent associations with health outcomes, has only been investigated in one study. To the best of our knowledge, whether the presence of PN is associated with lower levels of PA and more sedentary behaviour in persons with T2DM compared with those without remains unclear due to the scarcity of evidence. Therefore, the present study examined the independent and combined associations of T2DM and impaired VPT as a marker of PN with accelerometer‐based measures of PA and sedentary behaviour in daily life in a large observational study. We hypothesised that individuals with both T2DM and PN have the lowest levels of physical activity and highest sedentary time compared to individuals not having these conditions or having only T2DM or PN.

## 2 Materials and Methods

### 2.1 Study Population

We used data from The Maastricht Study, an observational prospective population‐based cohort study. The rationale and methodology have been described previously [21]. In brief, the study focuses on the aetiology, pathophysiology, complications and comorbidities of T2DM and is characterised using an extensive phenotyping approach. Eligible for participation were all individuals aged between 40 and 75 years and living in the southern part of the Netherlands. Participants were recruited through mass media campaigns and from the municipal registries and the regional Diabetes Patient Registry via mailings. Recruitment was stratified according to known T2DM status, with an oversampling of individuals with T2DM, for efficiency reasons. The present report includes cross‐sectional data from the first 9187 participants who completed the baseline survey between November 2010 and October 2020. Examinations of each participant were performed within a time window of three months. The study has been approved by the institutional medical ethical committee (NL31329.068.10) and the Minister of Health, Welfare and Sports of the Netherlands (Permit 131088‐105234‐PG). All participants gave written informed consent.

### 2.2 Impaired Vibration Perception

Impaired vibration perception in the foot was determined through the measurement of the VPT using a Horwell neurothesiometer (Scientific Laboratory Supplies, UK). The neurothesiometer's probe was gently placed on both halluces surfaces with the voltage being gradually increased by the examiner. The smallest voltage at which vibration is perceived by an individual is the VPT. The VPT measurement was repeated three times per foot and the outputs were averaged. If no vibration was felt even at the maximum device voltage, the output from the measurement was recorded as the maximum device voltage. An impaired VPT response is an early indicator in certain types of neuropathies, such as PN [22, 23]. Additionally, elevated VPT values have been linked to the presence of PN symptoms and are predictive of severe complications such as foot ulceration [24, 25]. A VPT exceeding 25 V in one or both halluces is indicative of impaired VPT [24]. Based on the VPT measurements and setting, the threshold at 25 V for dichotomisation, a new variable denoting impaired VPT referred to in this text as iVPT, was created for all participants.

### 2.3 Diabetes

Prevalent T2DM and prediabetes (preDM) were defined in accordance with WHO 2006 criteria [26]. All participants underwent a standardized oral glucose tolerance test after overnight fasting. Blood samples were collected at baseline and 120 min after the consumption of a 75‐g glucose drink. Participants who were insulin‐treated or had a fasting glucose level higher than 11.0 mmol/L (as determined by finger prick) did not undergo this test. Participants on diabetes medication were also considered to have T2DM. Other types of diabetes such as gestational diabetes and type 1 diabetes were excluded from this study.

### 2.4 Physical Activity and Sedentary Behaviour

Daily PA and sedentary behaviour were measured with the activPAL3 accelerometer (PAL Technologies, Glasgow, UK), a small (53 × 35 × 7 mm) light‐weight triaxial accelerometer as described in more detail elsewhere [27]. The accelerometer was waterproofed with a nitrile sleeve and attached to the right thigh during the first visit to the research centre. Participants were instructed to wear the device during 8 days consecutively and, in case of removal, the device was not replaced. Data from the first day were excluded from the analysis because participants performed physical function tests at the research centre after the device was attached. In addition, data of the final wear day providing < 14 hours of data were excluded from analysis. Data were uploaded using the activPAL software and processed using a customised algorithm written in MATLAB R2013b (MathWorks. Natick, MA, USA) that allowed the identification of wake‐time and in‐bed time [28]. Wake‐time was further differentiated in this study for PA and sedentary behaviour outcomes. PA outcomes included mean step count per day, mean standing time per day, minutes per day in light intensity physical activity (LIPA, stepping frequency < 100 steps/minute), minutes per day in moderate‐to‐vigorous physical activity (MVPA, stepping frequency ≥ 100 steps/minute). Sedentary time was based on sedentary position (sitting or lying) and calculated as the mean time spent in the sedentary position per day. Other sedentary behaviour outcomes included mean sedentary bout duration per day and the mean number of sedentary breaks per day, defined as interruptions in sedentary time lasting at least 1 min.

### 2.5 Covariates

Covariates were extracted from questionnaires and measurements carried out by an examiner during the visit to The Maastricht Study facility [21]. These included sociodemographic variables (age, sex and educational level) and health and behaviour related variables (smoking status, waist circumference, peripheral arterial disease and kidney disease). Educational level was categorised into low, medium and high, while smoking status was categorised into never, former and current. Peripheral arterial disease was defined when the measurement of the ankle brachial index was lower than 0.9 or greater than 1.3. Kidney disease was defined when either an estimated glomerular filtration rate was below 60 mL/min/1.73 $m^{2}$ with normal albumen excretion or elevated albumen excretion (> 30 mg/24 h) with normal estimated glomerular filtration rate, or when both were abnormal.

### 2.6 Statistical Analyses

Four groups were created based on the combination of the presence of T2DM (yes/no) and iVPT (yes/no). Baseline characteristics are presented for the defined groups. Continuous variables are shown as mean ± standard deviation. Categorical variables are shown as counts and percentages.

First, the independent associations of T2DM and iVPT with PA and sedentary behaviour variables were assessed using multiple linear regression analyses. Four models were created for adjusting for potential confounders: Model 1 was unadjusted; Model 2 was adjusted for age, sex and educational level; Model 3 was additionally adjusted for smoking status, waist circumference, peripheral arterial disease, and kidney disease and Model 4 was additionally adjusted for either T2DM (in the model with iVPT as the main independent variable) or iVPT (in the model with T2DM as the main independent variable).

Next, the combined association of T2DM and iVPT groups as exposure and PA and sedentary behaviour outcomes were analysed using multiple linear regression analyses with the group without any of the conditions (T2DM−/iVPT−) serving as the reference group. Model 1 was unadjusted; Model 2 was adjusted for age, sex and educational level and Model 3 was additionally adjusted for smoking status, waist circumference, peripheral arterial disease, and kidney disease. We also formally tested the interaction between T2DM and iVPT by introducing in the independent analysis the product term between T2DM and iVPT.

Several additional analyses were conducted. First, we checked for possible effect modification by sex, age and educational level. Next, waist circumference was replaced by body mass index as a confounder; educational level was replaced by income and occupational status and peripheral arterial disease was replaced by a history of cardiovascular disease. Finally, we added preDM (yes/no) as an independent variable in the combined analysis. This led to a total of six groups: normal glucose metabolism (NGM)/−iVPT, NGM/+iVPT, PreDM/−iVPT, PreDM/+iVPT, T2DM/−iVPT and T2DM/+iVPT. We assessed the combined associations between the six groups and PA/sedentary behaviour outcomes, with the group with none of the conditions (NGM/iVPT−) serving as the reference group.

All analysis were performed using SPSS 28 (IBM Corp, Armonk, NY, USA), and *p*‐values < 0.05 was considered statistically significant.

## 3 Results

**FIGURE 1:**
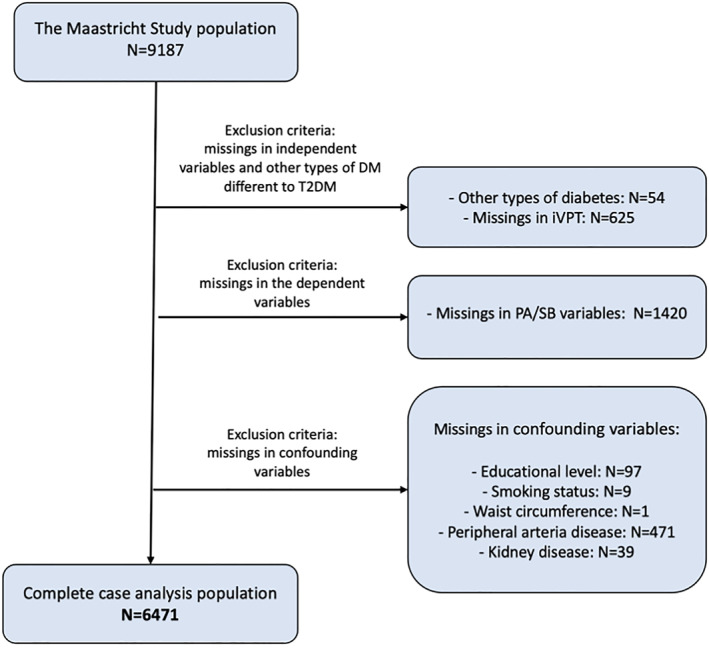
Flowchart for the study population. The flowchart shows the number of missing values per variable of interest and the exclusion of participants with other types of diabetes. A considerable amount of missing data for PA/sedentary behaviour is due to the fact that this measurement was introduced approximately 9 months after baseline data collection had started.

**FIGURE 2:**
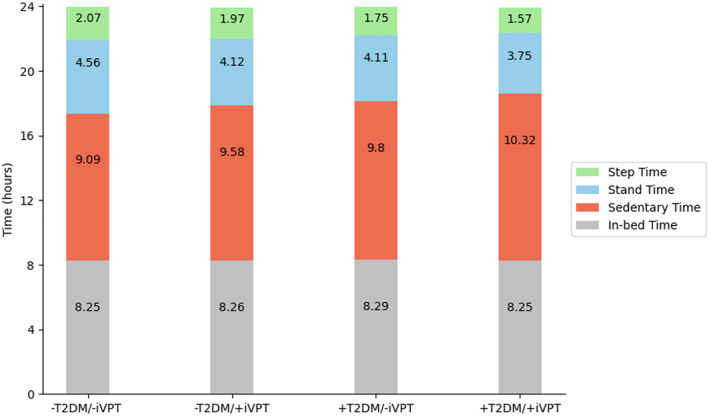
24‐h behaviour distribution of stepping time, standing time, sedentary time and in‐bed time according to T2DM and iVPT status. Each bar represents the average time in hours spent in stepping, standing, sedentary and in‐bed activities according to T2DM and iVPT status. Participants with both T2DM and iVPT showed the lowest step time and highest sedentary time.

Population characteristics can be found in Table 1. Figure 1 shows a flow chart detailing the number of participants with missing variables and the final complete case analysis sample, which included 6471 participants. In our analysis sample (Table 1), 4618 participants had no T2DM and no iVPT (T2DM−/iVPT−), 479 participants had iVPT and no T2DM (T2DM−/iVPT+), 1087 had T2DM and no iVPT (T2DM+/iVPT−), and 287 had both T2DM and iVPT (T2DM+/iVPT+). Therefore, the prevalence of iVPT was 9.4% in the group without T2DM and 20.9% in the group with T2DM, while the prevalence of T2DM in individuals with iVPT was 37.5% and 19.1% in those without iVPT. Individuals within the T2DM+/iVPT+ group were more often men, had lower educational levels and were more often former smokers. The 24‐h activity analysis showed that individuals with T2DM+/iVPT+ presented more time in sedentary behaviour and less stepping time over the day compared to the rest of the groups (Figure 2).

**TABLE 1:** Descriptive characteristics.

		T2DM−/iVPT−(*N* = 4618, 71.4%)	T2DM−/iVPT+ (*N* = 479, 7.4%)	T2DM+/iVPT−(*N* = 1087, 16.8%)	T2DM+/iVPT+ (*N* = 287, 4.4%)
Age (years)		58.1 ± 8.7	65.8 ± 6.3	62.3 ± 7.9	66.9 ± 6.4
Sex	Male	1958 (42.4%)	331 (69.1%)	685 (63.0%)	240 (83.6%)
	Female	2660 (57.6%)	148 (30.9%)	402 (37.0%)	47 (16.4%)
Waist circumference (cm)		91.0 ± 11.7	97.3 ± 12.2	104.8 ± 12.3	108.7 ± 12.6
Body mass index ($\mathrm{kg}/m^{2}$)		25.9 ± 3.8	26.5 ± 3.8	29.4 ± 4.5	29.9 ± 4.5
Height (cm)		170.2 ± 9.0	176.0 ± 9.0	170.4 ± 8.8	175.1 ± 8.3
Smoking status	Never	1947 (42.0%)	161 (33.6%)	361 (33.2%)	67 (23.3%)
	Former	2134 (46.2%)	257 (53.7%)	557 (51.2%)	186 (64.8%)
	Current	543 (11.8%)	61 (12.7%)	169 (15.6%)	34 (11.9%)
Educational level	Low	1339 (29.0%)	191 (39.9%)	481 (44.3%)	132 (46.0%)
	Medium	1275 (27.6%)	115 (24.0%)	298 (27.4%)	82 (28.6%)
	High	2004 (43.4%)	173 (36.1%)	308 (28.3%)	73 (25.4%)
VPT right hallux (V)		10.1 ± 5.1	28.2 ± 7.9	12.0 ± 5.4	30.2 ± 9.3
VPT left hallux (V)		10.1 ± 5.2	27.8 ± 7.3	11.8 ± 5.4	30.9 ± 8.6
Step count (steps/day)		10,090 ± 3655	9478 ± 3885	8322 ± 3478	7346 ± 3480
LIPA (min/day)		340.6 ± 91.9	314.2 ± 90.3	306.5 ± 94.4	282.0 ± 99.6
MVPA (min/day)		56.7 ± 25.4	51.0 ± 26.8	44.8 ± 23.7	37.2 ± 23.7
Sedentary time (min/day)		545.3 ± 96.7	575.0 ± 101.9	588.2 ± 103.3	619.5 ± 106.1
Sedentary bout duration (min/bout)		10.6 ± 3.2	11.8 ± 3.8	12.2 ± 4.0	13.3 ± 4.1
Sedentary breaks (breaks/day)		38.1 ± 8.5	36.5 ± 8.2	36.20 ± 8.8	34.8 ± 8.0

*Note:* Continuous variables are presented by their mean and standard deviation while categorical variables are represented as a count and percentage within the group.

The independent associations of T2DM and iVPT with PA and sedentary behaviour are reported in Table 2. Based on Model 3, individuals with T2DM took, in comparison to those without T2DM, 8.5% fewer steps per day (−837 [95% CI: −1069, −605]), spent less time standing (−10.4 min/day [−15.5, −5.2]), and accumulated less time in LIPA (−15.3 min/day [−21.2, −9.3]) and MVPA (−5.0 min/day [−6.6, −3.4]). Furthermore, individuals with T2DM exhibited more sedentary time (+18.5 min/day [12.3, 24.7]) and sedentary bouts lasting longer (+0.5 min/bout [0.3, 0.8]) than individuals without T2DM (Table 2—**Model 3**). After adjusting Model 4 for iVPT, the results did not materially change (Table 2—**Model 4**).

**TABLE 2:** Independent association of T2DM and iVPT with PA and sedentary behaviour outcomes.

Independent analysis: Main independent variable is T2DM				
	Model 1	Model 2	Model 3	Model 4: Model 3 + iVPT
	B [95% CI]	B [95% CI]	B [95% CI]	B [95% CI]
Step count (steps/day)	−1912 [−2129, −1694]	−1723 [−1949, −1497]	−837 [−1069, −605]	−828 [−1060, −596]
Standing time (min/day)	−28.9 [−33.7, −24.1]	−20.3 [−21.2, −15.5]	−10.4 [−15.5, −5.2]	−10.0 [−15.2, −4.9]
LIPA (min/day)	−36.7 [−42.2, −31.2]	−28.4 [−34.0, −22.8]	−15.3 [−21.2, −9.3]	−14.9 [−20.9, −9.0]
MVPA (min/day)	−13.0 [−14.5, −11.5]	−10.9 [−12.5, −9.4]	−5.0 [−6.6, −3.4]	−4.9 [−6.5, −3.3]
Sedentary time (min/day)	46.6 [40.7, 52.5]	36.4 [30.5, 42.3]	18.5 [12.3, 24.7]	18.1 [11.9, 24.4]
Sedentary bout duration (min/bout)	1.7 [1.5, 1.9]	1.3 [1.1, 1.5]	0.5 [0.3, 0.8]	0.5 [0.3, 0.7]
Sedentary breaks (breaks/day)	−2.1 [−2.6, −1.5]	−1.4 [−2.0, −0.9]	−0.4 [−0.9, 0.2]	−0.4 [−0.9, 0.2]

Independent analysis: Main independent variable is iVPT				
	Model 1	Model 2	Model 3	Model 4: Model 3 + T2DM
	B [95% CI]	B [95% CI]	B [95% CI]	B [95% CI]
Step count (steps/day)	−1070 [−1350, −790]	−704 [−996, −412]	−356 [−635, −77]	−322 [−600, −44]
Standing time (min/day)	−29.6 [−35.7, −23.5]	−15.5 [−21.8, −9.3]	−11.8 [−18.0, −5.6]	−11.4 [−17.5, −5.2]
LIPA (min/day)	−31.9 [−39.0, −24.9]	−17.2 [−24.4, −10.0]	−12.1 [−19.2, −4.9]	−11.5 [−18.6, −4.4]
MVPA (min/day)	−8.7 [−10.6, −6.7]	−5.5 [−7.5, −3.5]	−3.2 [−5.1, −1.3]	−3.0 [−4.9, −1.1]
Sedentary time (min/day)	38.2 [30.6, 47.7]	20.0 [12.2, 27.5]	12.9 [5.5, 20.4]	12.2 [4.7, 19.6]
Sedentary bout duration (min/bout)	1.4 [1.1, 1.7]	0.7 [0.4, 1.0]	0.4 [0.2, 0.7]	0.4 [0.1, 0.7]
Sedentary breaks (breaks/day)	−1.9 [−2.5, −1.2]	−0.9 [−1.5, −0.2]	−0.5 [−1.2, 0.2]	−0.5 [−1.2, 0.2]

*Note:* Model 1 was unadjusted; Model 2 was adjusted for age, sex and educational level; Model 3 was adjusted for age, sex, educational level, smoking status, waist circumference, peripheral arterial disease, and kidney disease; and Model 4 was additionally adjusted either for T2DM or iVPT.

Compared to those without iVPT, individuals with iVPT took 3.7% fewer steps (−356 steps/day [−635, −77], spent fewer minutes per day in standing (−11.8 min/day [−18.0, −5.6]), and accumulated fewer minutes in LIPA (−12.1 min/day [−19.2, −4.9]) and MVPA (−3.2 min/day [−5.1, −1.3]) (Table 2—**Model 3**). Moreover, those with iVPT exhibited more sedentary time (+12.9 min/day [5.5, 20.4]) and had longer sedentary bouts (+0.4 min/bout [0.2, 0.7]) compared with those without iVPT. After further adjustment for T2DM in Model 4, the results remained similar (Table 2—**Model 4**).

In the combined analysis, individuals with T2DM+/iVPT+ showed the lowest (−14.2%) step count (−1407 steps/day [−1851, −963]), compared to the reference group. The T2DM+/iVPT+ group also had the lowest time standing (−20.8 min/day [−30.7, −11.0]), time in LIPA (−27.2 min/day [−38.6, −15.8]) and in MVPA (−9.5 min/day [−12.6, −6.5]), compared to the reference group (Table 3—**Model 3**). The combined presence of T2DM and iVPT was also associated with the most sedentary time (+33.3 min/day [21.4, 45.2]) and longest sedentary bouts (+1.0 min/bout [0.6, 1.4]).

**TABLE 3:** Combined association between T2DMand iVPT status and PA and sedentary behaviour outcomes.

Combined analysis: T2DM and iVPT status				
		Model 1	Model 2	Model 3
		B [95% CI]	B [95% CI]	B [95% CI]
Step count (steps/day)	T2DM−/iVPT−	REF	REF	REF
	T2DM−/iVPT+	−612 [−954, −2670]	−395 [−748, −41]	−132 [−473, 209]
	T2DM+/iVPT−	−1768 [−2008, −1527]	−1612 [−1860, −1364]	−733 [−985, −481]
	T2DM+/iVPT+	−2734 [−3167, −2300]	−2472 [−2920, −2024]	−1407 [−1851, −963]
Standing time (min/day)	T2DM−/iVPT−	REF	REF	REF
	T2DM−/iVPT+	−26.3 [−33.9, −18.8]	−14.5 [−22.1, −6.9]	−11.8 [−19.4, −4.9]
	T2DM+/iVPT−	−26.9 [−32.2, −21.6]	−19.9 [−25.2, −14.6]	−10.2 [−15.8, −4.7]
	T2DM+/iVPT+	−48.6 [−58.2, −39.0]	−32.6 [−42.2, −22.9]	−20.8 [−30.7, −11.0]
LIPA (min/day)	T2DM−/iVPT−	REF	REF	REF
	T2DM−/iVPT+	−26.4 [−35.1, −17.7]	−14.5 [−23.3, −5.7]	−10.9 [−19.6, −2.2]
	T2DM+/iVPT−	−34.1 [−40.2, −27.9]	−27.5 [−33.7, −21.3]	−14.6 [−21.1, −8.2]
	T2DM+/iVPT+	−58.6 [−69.6, −47.5]	−42.8 [−54.0, −31.6]	−27.2 [−38.6, −15.8]
MVPA (min/day)	T2DM−/iVPT−	REF	REF	REF
	T2DM−/iVPT+	−5.8 [−8.2, −3.4]	−3.5 [−6.0, −1.1]	−1.8 [−4.2, 0.6]
	T2DM+/iVPT−	−11.9 [−13.6, −10.2]	−10.2 [−11.9, −8.5]	−4.3 [−6.1, −2.6]
	T2DM+/iVPT+	−19.6 [−22.6, −16.6]	−16.6 [−19.7, −13.5]	−9.5 [−12.6, −6.5]
Sedentary time (min/day)	T2DM−/iVPT−	REF	REF	REF
	T2DM−/iVPT+	29.7 [20.4, 39.0]	15.1 [5.8, 24.4]	10.0 [0.9, 19.1]
	T2DM+/iVPT−	42.8 [36.3, 49.4]	34.7 [28.2, 41.2]	17.1 [10.3, 23.8]
	T2DM+/iVPT+	74.13 [62.4, 85.9]	54.6 [42.8, 66.4]	33.3 [21.4, 45.2]
Sedentary bout duration (min/bout)	T2DM−/iVPT−	REF	REF	REF
	T2DM−iVPT+	1.1 [0.8, 1.5]	0.6 [0.2, 0.9]	0.4 [0.0, 0.7]
	T2DM+/iVPT−	1.6 [1.4, 1.8]	1.2 [1.0, 1.5]	0.5 [0.3, 0.7]
	T2DM+/iVPT+	2.6 [2.3, 3.1]	1.9 [1.5, 2.3]	1.0 [0.6, 1.4]
Sedentary breaks (breaks/day)	T2DM−/iVPT−	REF	REF	REF
	T2DM−/iVPT+	−1.6 [−2.4, −0.8]	−0.7 [−1.5, 0.2]	−0.4 [−1.2, 0.4]
	T2DM+/iVPT−	−1.9 [−2.5, −1.4]	−1.4 [−1.9, −0.8]	−0.3 [−0.9, 0.3]
	T2DM+/iVPT+	−3.3 [−4.3, −2.3]	−2.3 [−3.3, −1.2]	−1.0 [−2.0, 0.1]

*Note:* Model 1 was unadjusted; Model 2 was adjusted for age, sex and educational level; Model 3 was adjusted for age, sex, educational level, smoking status, waist circumference, peripheral arterial disease, and kidney disease; and Model 4 was additionally adjusted either for T2DM or iVPT.

The formal test for interaction analysis between T2DM and iVTP did not reveal any statistically significant interactions for the outcomes (all *p*‐values > 0.05, Supporting Information S1: Table 1). The results for the additional analyses, where covariates were replaced for other variables of interest, are reported in the Supporting Information S1: Table 2. When body mass index replaced waist circumference in the combined analysis, Model 3 showed lower PA levels and higher sedentary time for individuals with T2DM+/iVPT+ compared with the initial findings for T2DM+/iVPT+ with waist circumference as a confounder. When income replaced educational level as a confounding variable, individuals with T2DM+/iVPT+ exhibited higher levels of PA and slightly lower sedentary time compared to the initial findings for T2DM+/iVPT+ with educational level as a confounder. Finally, when history of cardiovascular disease replaced peripheral arterial disease, individuals with T2DM+/iVPT+ presented similar levels of PA and sedentary time as shown in the main results with peripheral arterial disease as a confounder.

The independent and combined associations of NGM, preDM, T2DM and iVPT showed that individuals with either preDM, T2DM or iVPT presented lower levels of PA and higher levels of sedentary time compared to the NGM/iVPT‐reference group (Supporting Information S1: Table 3—**Model 3**). Individuals with preDM/iVPT‐ took fewer steps (−295 steps/day [−565, −26]) and accumulated more sedentary time (+6.3 min/day [−0.9, 13.5]) compared to the reference group. Levels of PA and sedentary time in those with NGM/iVPT+ did not significantly differ from those in the NGM/iVPT‐group (*p* > 0.05). However, individuals with preDM+/iVPT+ showed lower step count (−398 steps/day [−1020, 223]) and more sedentary time (+22.3 min/day [5.7, 38.9]) compared to individuals without any of the conditions (NGM/iVPT−).

## 4 Discussion

In this large cohort study, we examined the independent and combined associations of T2DM and iVPT with device‐measured PA and sedentary behaviour. We showed that both the presence of T2DM and the presence of PN were independently associated with lower step count, fewer minutes in standing, LIPA and MVPA, and more sedentary time with longer sedentary bouts. Moreover, the combination of T2DM and PN was associated with the lowest step count, lowest time in both LIPA and MVPA, and highest sedentary time, compared to those without any of the conditions.

Both our findings and previous studies have shown that T2DM is associated with low PA measures such as daily step count, LIPA and MVPA and more sedentary time [27, 29]. The present study extends those data by investigating PA outcomes in individuals with T2DM and PN as well as in those with only PN (and no diabetes). To date, only one study has separately looked at sedentary behaviour but did not find a significant association between PN and sedentary time in people with diabetes [18]. However, this study [18] showed that older individuals without diabetes with worse sensory nerve action potential amplitude had 2.2 min/day less in self‐reported vigorous PA. This is in line with our study as we found that individuals without T2DM and with iVPT engage in 1.8 min/day less in MVPA. In another study, individuals with T2DM and self‐reported PN had lower levels of PA than those without PN, using questionnaires to measure PA [19]. Using objectively reported PA, a study investigating daily walking activity with a pedometer reported that in individuals with T2DM, the presence of PN was associated with 1967 fewer steps/day [20]. These findings concur with the current study where we showed that the coexistence of T2DM and iVPT was associated with 1407 fewer steps/day, whereas the presence of T2DM alone was associated with 828 fewer steps/day.

Several factors may explain the higher levels of sedentary behaviour and lower levels of PA in those with T2DM compared with individuals without diabetes. These include comorbidities such as obesity and cardiovascular disease, poor T2DM self‐management efficacy or psychological factors such as depression or low levels of motivation [30, 31, 32]. Additionally, specific biomechanical conditions have been associated with lower levels of PA in individuals with T2DM, such as reduced ankle joint torque and muscle weakness [33]. Also, a recent meta‐analysis has shown that individuals with T2DM and PN have a longer stride duration while walking, and this could explain the lower step count in those with T2DM and PN [34].

Our results show that the coexistence of PN with T2DM further aggravates the relationship between PA and sedentary time. Sensory alterations are primary characterised by the loss of sensation and altered proprioceptive feedback from the lower extremities [9], which in turn can lead to impaired balance, altered gait patterns and reduced walking stability [9, 11]. Consequently, PN may have an impact on individuals' daily physical activity levels and sedentary time, potentially increasing the risk of mobility disability. Future research is needed to further examine the impact of T2DM and PN on physical function and to what extent this is mediated by PA and sedentary behaviour. It should be noted that PN in our cohort was not only prevalent in persons with T2DM (almost 21%) but also in those without diabetes (9%). Future research should also examine the health consequences of PN in the general population.

While PN symptoms and sensorimotor impairments increase the difficulty of engaging in PA, PN symptoms may be improved with PA interventions. A recent systematic review [35] concluded that PA is beneficial for individuals with PN. Specifically, some studies have shown that PA reduces PN symptoms, improves gait performance and disrupts the normal progression of PN in individuals with diabetes [17, 36, 37]. Additionally, Loprinzi et al. showed in a cross‐sectional study in persons with diabetes that those who were more physically active, coupled with glycaemic control, were less likely to have PN [38]. This suggests that PA may protect against the progression of PN. To further study this relationship, longitudinal research is needed, and it may help define strategies capable of reversing to some extent PN and its consequences for mobility.

### 4.1 Strengths and Limitations

A main strength of this study is the large population sample size, which increases statistical power, and the oversampling of individuals with T2DM, ensuring adequate representation of both T2DN and iVPT. In this study, 6471 participants were included, whereas similar studies reporting PA in individuals with and without T2DM and PN included 328 participants [18], 481 participants [19] and 100 participants [20]. Another strength is the use of device‐based measures of PA and sedentary behaviour. Self‐reporting PA is subjected to recall and reporting bias [39]. Additionally, the use of accelerometers allows the description of several types of daily behaviour from sedentary time to activities at different intensities. From the few studies that investigated PA in individuals with PN [18, 19, 20], only one of them used device‐based measures of PA but was limited to step count as a pedometer was used instead of an accelerometer. Another strength is the correction for several confounders that could have influenced our results. A limitation of this study is its cross‐sectional nature; therefore, we cannot imply causation. Further, assessing PN is challenging due to the different signs and symptoms shown by patients and the complexity or subjectivity of measurement tools and methods used for the diagnosis of PN. In daily practice, Semmes–Weinstein monofilaments, which assess pressure sensation and predict future ulceration, are frequently used to screen test for PN [40]. In this large scale study, we used VPT testing as a measure of sensory nerve function for several reasons, including the relation of proprioception with mobility, its acceptable reliability and diagnostic accuracy for determining the presence of PN and its ease to standardise when used by multiple examiners [41, 42]. Compared to other objective measures of nerve function, such as nerve conduction studies, the use of a neurothesiometer can be seen as a limitation as it relies on patient's response and, additionally, the test does not inform about motor nerve function. However, other tests such as nerve conduction studies present other challenges such as data reproducibility and interpretation. Another limitation of this study is the use of a threshold for identifying iVPT. The 25 V used as threshold has been linked to serious PN complications and symptoms [23] but may not identify individuals with less severe iVPT. Also, symptoms of PN were not assessed. In future studies, both sensory and motor peripheral nerve function should be studied in more detail, and how these functions are associated with PA and sedentary behaviour. Future longitudinal studies assessing how changes in VPT, as a marker of nerve damage, relate over time to PA and sedentary behaviour levels are also needed to further understand the associations between PN and PA levels.

### 4.2 Conclusions

In this cohort, PN was a relatively prevalent condition in middle‐aged and older adults without diabetes and—as expected—its prevalence was markedly increased in those with T2DM. T2DM and PN were both independently associated with lower levels of both light and moderate‐vigorous PA and higher levels of sedentary behaviour. The coexistence of T2DM and PN was associated with particularly low levels of PA and higher levels of sedentary time indicating an additive association. Clinicians should be aware that individuals with T2DM and PN are particularly prone to being physically inactive. PA interventions in these individuals should take the limitations caused by the PN into account as well as the increased risk of developing a foot ulcer that may be the consequence of such interventions. Further research is needed to understand how to design intervention strategies for this group of individuals.

## Author Contributions

T.A., A.K., H.S., N.S. and B.B. contributed to conception and design of the study. T.A. ran the statistical analysis and wrote the draft manuscript. T.A., A.K., H.S., N.S., B.B., H.B., M.v.G., B.d.G. and M.T.S. contributed to interpretation of the literature and results, and revising the manuscript. All authors gave final approval of the version for publication.

## Conflicts of Interest

The authors declare no conflicts of interest.

## Peer Review

The peer review history for this article is available at https://www.webofscience.com/api/gateway/wos/peer-review/10.1002/dmrr.70069.

## Supporting information

Supporting Information S1

## Data Availability

The data of this study derive from The Maastricht Study, but restrictions apply to the availability of the data which were used under licence for the current study. Data are, however, available from the authors upon reasonable request and with permission of The Maastricht Study management team.
