# Supplementary material for: Combined Associations of Type 2 Diabetes and Peripheral Neuropathy With Device‐Measured Physical Activity and Sedentary Behaviour—The Maastricht Study
Source: Diabetes Metab Res Rev. 2025 Jul 22;41(5):e70069. doi: 10.1002/dmrr.70069 (PMC12284459; doi:10.1002/dmrr.70069)
Supplement: Supplementary file 1 — Supporting Information S1 [file DMRR-41-e70069-s001.docx]

**Supplementary materials**

**Supplementary Table 1: Interaction term between T2DM and iVPT.**

| **T2DM and iVPT interaction term** | | |
| --- | --- | --- |
| **Interaction Term** | **Independent Variables** | **p-value** |
| **T2DM*iVPT** | **Step Count (steps/day)** | 0.06 |
|  | **Standing Time (min/day)** | 0.83 |
|  | **LIPA (min/day)** | 0.83 |
|  | **MVPA (min/day)** | 0.08 |
|  | **Sedentary Time (min/day)** | 0.42 |
|  | **Sedentary Time (min/day)** | 0.42 |
|  | **Total Sedentary Breaks (breaks/day)** | 0.62 |

Results for the interaction terms are presented as p-values. Analyses were adjusted for age, sex, educational level, smoking status, waist circumference, peripheral arterial disease, and kidney disease.

**Supplementary Table 2:** Additional analysis with replacement for confounding variables.

| **Combined Analysis:** T2DM+/iVPT+ outcomes compared to the reference group (T2DM-/iVPT-) | | | |
| --- | --- | --- | --- |
|  | **Model 3:** body mass index replaced waist circumference. | **Model 3:** income replaced educational level. | **Model 3:** history of cardiovascular disease replaced peripheral arterial disease. |
|  | **B [95% CI]** | **B [95% CI]** | **B [95% CI]** |
| **Step Count (steps/day)** | -1701  [-2145, -1257] | -1193  [-1722, -665] | -1341  [-1786, -896] |
| **Standing Time (min/day)** | -27.2  [-37.0, -17.4] | -25.8  [-37.4, -14.1] | -20.7  [-30.6, -10.8] |
| **LIPA**  **(min/day)** | -34.6  [-46.0, -23.2] | -32.0  [-45.5, -18.5] | -26.8  [-38.2, -15.4] |
| **MVPA**  **(min/day)** | -11.5  [-14.6, -8.4] | -7.3  [-11.0, -3.7] | -9.1  [-12.2, -6.0] |
| **Sedentary Time (min/day)** | 41.3  [29.4, 53.2] | 32.1  [17.9, 46.2] | 33.0  [21.1, 45.0] |
| **Sedentary Bout Duration (min/bout)** | 1.2  [0.8, 1.6] | 1.1  [0.6, 1.6] | 0.1  [0.5, 1.4] |
| **Sedentary Breaks (breaks/day)** | -1.2  [-2.3, -0.2] | -1.1  [-2.3, 0.2] | -0.9  [-1.9, 0.2] |

Model 3 was initially adjusted for age, sex, educational level, smoking status, waist circumference, peripheral arterial disease and kidney disease. The additional analysis replaced waist circumference by body mass index, educational level by income and peripheral arterial disease by history of cardiovascular disease.

**Supplementary Table 3:** Combined association between NGM, PreDM, T2DMand iVPT with PA and sedentary behaviour outcomes.

| **Combined analysis**: NGM, PreDM, T2DM and iVPT | | **Model 3** |
| --- | --- | --- |
|  |  | **B [95% CI]** |
| **Step Count (steps/day)** | **NGM/iVPT-** (N=3779) | REF |
|  | **NGM/iVPT+** (N=347) | -137 [-532, 258] |
|  | **preDM+/iVPT-** (N=839) | -295 [-565, -26] |
|  | **preDM+/iVPT+** (N=132) | -398 [-1020, 223] |
|  | **T2DM+/iVPT-** (N=1087) | -817 [-1080, -554] |
|  | **T2DM+/iVPT+** (N=287) | -1507 [-1960, -1054] |
| **Standing Time (min/day)** | **NGM/iVPT-** | REF |
|  | **NGM/iVPT+** | -7.9 [-16.6, 0.9] |
|  | **preDM+/iVPT-** | 0.9 [-5.1, 6.9] |
|  | **preDM+/iVPT+** | -21.8 [-35.5, -8.0] |
|  | **T2DM+/iVPT-** | -10.2 [-16.0, -4.4] |
|  | **T2DM+/iVPT+** | -20.8 [-30.8, -10.8] |
| **LIPA**  **(min/day)** | **NGM/iVPT-** | REF |
|  | **NGM/iVPT+** | -6.5 [-16.6, 3.6] |
|  | **preDM+/iVPT-** | 0.7 [-6.2, 7.6] |
|  | **preDM+/iVPT+** | -22.1 [-38.0, -6.2] |
|  | **T2DM+/iVPT-** | -14.7 [-21.4, -7.9] |
|  | **T2DM+/iVPT+** | -27.3 [-38.9, -15.7] |
| **MVPA**  **(min/day)** | **NGM/iVPT-** | REF |
|  | **NGM/iVPT+** | -1.9 [-4.6, 0.8] |
|  | **preDM+/iVPT-** | -2.1 [-4.0, -0.3] |
|  | **preDM+/iVPT+** | -3.5 [-7.8, 0.8] |
|  | **T2DM+/iVPT-** | -4.9 [-6.7, -3.1] |
|  | **T2DM+/iVPT+** | -10.2 [-13.4, -7.1] |
| **Sedentary Time (min/day)** | **NGM/iVPT-** | REF |
|  | **NGM/iVPT+** | 7.7 [-2.9, 18.2] |
|  | **preDM+/iVPT-** | 6.3[-0.9, 13.5] |
|  | **preDM+/iVPT+** | 22.3 [5.7, 38.9] |
|  | **T2DM+/iVPT-** | 19.0 [11.9, 26.0] |
|  | **T2DM+/iVPT+** | 35.6 [23.5, 47.7] |
| **Sedentary Bout Duration (min/bout)** | **NGM/iVPT-** | REF |
|  | **NGM/iVPT+** | 0.1 [-0.2, 0.5] |
|  | **preDM+/iVPT-** | -0.1 [-0.3, 0.2] |
|  | **preDM+/iVPT+** | 0.9 [0.3, 1.5] |
|  | **T2DM+/iVPT-** | 0.5 [0.3, 0.7] |
|  | **T2DM+/iVPT+** | 1.0 [0.6, 1.4] |
| **Sedentary Breaks (breaks/day)** | **NGM/iVPT-** | REF |
|  | **NGM/iVPT+** | -0.0 [-0.9, 0.9] |
|  | **preDM+/iVPT-** | 0.4 [-0.2, 1.0] |
|  | **preDM+/iVPT+** | 1.1 [-2.6, 0.4] |
|  | **T2DM+/iVPT-** | 0.2 [-0.9, 0.4] |
|  | **T2DM+/iVPT+** | 0.9 [-1.9, 0.2] |

Analyses adjusted for age, sex, educational level, smoking status, waist circumference, peripheral arterial disease and kidney disease.
